# Bioaccumulation of Antimony and Arsenic in Vegetables and Health Risk Assessment in the Superlarge Antimony-Mining Area, China

**DOI:** 10.1155/2015/909724

**Published:** 2015-09-09

**Authors:** Defang Zeng, Saijun Zhou, Bozhi Ren, Tengshu Chen

**Affiliations:** ^1^School of Resource and Environmental Engineering, Wuhan University of Technology, Wuhan 430070, China; ^2^College of Civil Engineering, Hunan University of Science and Technology, Xiangtan 411201, China

## Abstract

Heavy metal pollution in soils caused by mining and smelting has attracted worldwide attention for its potential health risks to residents. This paper studies the concentrations and accumulations of Sb and As in both soils and vegetables and the human health risks of Sb and As in vegetables from Xikuangshan (XKS) Sb mine, Hunan, China. Results showed that the soils were severely polluted by Sb and As; Sb and As have significant positive correlation. Sb and As concentrations in vegetables were quite different:* Coriandrum sativum* L. was the highest in Sb,* Allium fistulosum* L. was the highest in As, and* Brassica pekinensis* L. was the lowest in both Sb and As;* Daucus carota* L. and* Coriandrum sativum* L. showed advantage in accumulating Sb and As;* Coriandrum sativum* L. had higher capacity of redistributing Sb and As within the plant. Health risk assessment results showed that the hazard quotient (HQ) values of Sb and As in vegetables were in the ranges of 1.61–3.33 and 0.09–0.39, respectively; the chronic daily intake (CDI) and hazard quotient (HQ) values of Sb were over the safe limit recommended by FAO and WHO, indicating that long-term consumption of vegetables from the surrounding soils of XKS mine may bring health risks to residents.

## 1. Introduction

Antimony (Sb), a nonessential toxic metal for life, widely exists in the lithosphere [1–3]. Microamount of Sb entering the human body will cause diseases to liver, skin, respiratory and cardiovascular systems, or even cancer [4–6]. Like arsenic (As), Sb and its compounds are listed as pollutants of priority interest by the United States Environment Protection Agency [7] and the European Union [8]. However, compared with the widespread studies on As, Sb has drawn relatively little attention [9, 10].

China is the world's largest antimony producer, and its average annual production of antimony accounts for 80% of the global produce [11]. Xikuangshan (XKS) in Hunan, the world's largest Sb mine, is reported to produce 25% of the world's Sb. XKS mine has been under mining and smelting for over 110 years, and the longtime large-scale mining and smelting operations caused severe As and Sb pollution to the local environment [12–14]. Generally, the concentrations of Sb and As are usually less than 10 mg kg^−1^ [3, 15] in the nonpolluted soil, but they reach several thousand mg kg^−1^ and several hundred mg kg^−1^ in the mining areas (especially the areas near waste-rock piles, tailing piles, and smelters), respectively. Therefore, the plants grown around Sb mine accumulate high concentrations of Sb and As. For example,* Agrostis capillaris* L. grown around an Sb mine in Ribes Valley (Eastern Pyrenees) has a concentration of Sb and As of 68 mg kg^−1^ and 240 mg kg^−1^ in its buds [16]. Vegetables contain the vital elements for human health such as carbohydrates, protein, vitamins, fiber, and minerals, so they are people's essential dietary food [17]. However, the consumption of vegetables contaminated by heavy metals will impair human health severely [18]. In recent years, scholars had conducted studies on distribution, deposition of Sb and As in soils, bioavailability, health risk assessment, and concentration of Sb and As in plants around the Sb mines [19, 20], but few studies are concerned with the contamination of Sb and As in the soil-vegetable system and its health risk assessment.

Therefore, this study takes the soil-vegetable system around XKS Sb mine as the object and probes into the concentration of Sb and As in different parts of vegetables and assesses its health risks, aiming at providing guidance for the local residents to take safe vegetable consumption.

## 2. Materials and Methods

### 2.1. Study Area

XKS Sb mine, known as the “World's Antimony Capital,” is located in Lengshuijiang city, Hunan province, in central China (E111°18′57′′~E111°36′40′′, N27°30′49′′~27°50′38′′). It borders Lianyuan city in the east, Xinshao County in the south, and Xinhua County in the west and north, with a mining area of 70 km^2^ and an Sb reserve of 400,000 tons. XKS Sb mine has two parts: the South mine (Wuhua, Feishuiyan) and the North mine (Laokuangshan, Tongjiayuan). The ores are simple in composition, with stibnite as the ore mineral. The gangue minerals include quartz and calcite, with minor amounts of fluorite, barite, and secondary gypsum. The alteration of the host limestone is dominated by silicification and subordinately by carbonatization and, to a lesser extent, by baritization and fluoritization [21]. Lengshuijiang has the continental monsoon climate in subtropical zone, with the annual average temperature of 16–17.3°C, annual average rainfall of 1354 mm, and annual average relative humidity of 53.1%. In January, the mean temperature is 4.9°C, and in July the mean temperature is 28.2°C.

### 2.2. Sample Collection

**Figure 1:**
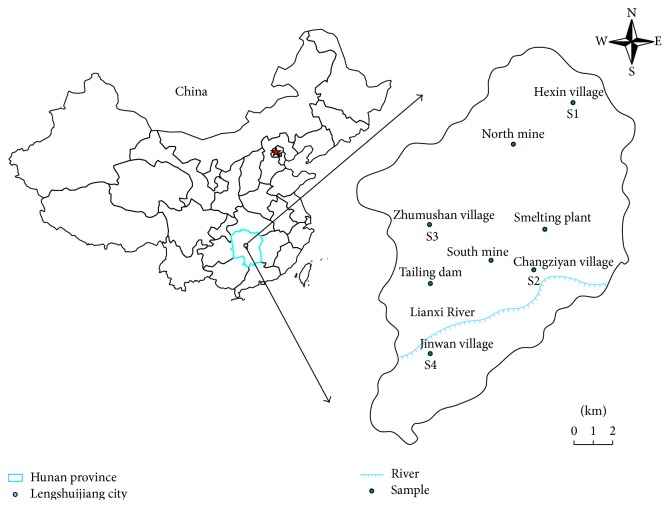
Map of sampling sites location.

The soil and vegetable samples for test were collected from the vicinity of XKS Sb mine (Figure 1) in January 2015. Four sampling sites (S1–S4) were selected for this study: S1 is located in Hexin village, about 2.0 km away from the North mine; S2 is located in Changziyan village, less than 1.0 km away from the waste-rock piles in the South mine and the smelter; S3 is located in Zhumushan village, about 0.5 km away from the tailings dam; S4 is located in Jinwan village at the downstream of Lian River, about 3.5 km away from South mine. Specimens of the vegetable species were collected together with their corresponding soils. Totally, 60 soil samples and 60 vegetable samples of 10 species were collected, with 15 soils samples and 15 vegetables collected at each sampling site. The 10 vegetable species are as follows:* Brassica pekinensis* L. (BP),* Brassica juncea* L. (BJ),* Allium fistulosum* L. (AF),* Allium sativum* L. (AS),* Raphanus sativus* L. (RS),* Daucus carota* L. (DC),* Coriandrum sativum* L. (CS),* Spinacia oleracea* L. (SO),* Chrysanthemum coronarium* L. (CC), and* Lactuca sativa* L. (LS). Samples were collected in accordance with plum-point sampling method, which means that a vegetable sample together with their corresponding soil sample at the plough layer (0–20 cm depth) is collected at the center and its four equidistant points of the vegetable plot. The soils were collected with a stainless steel spatula and then put into the PTFE plastic sampling bags after being well mixed. The samples were sent to the laboratory for processing the same day.

### 2.3. Sample Treatment and Analytical Methods

Once in the laboratory, the vegetables were washed with tap water to remove the surface soil and rinsed with ultrasonic cleaning to further remove the residual soil and finally washed three times with ultrapure water (18.0 MΩ·cm) to remove pesticides and contaminants. All aboveground parts of vegetables were separated from the underground parts. When there was no water left on the vegetables, they were dried in an oven at 45°C until they reached constant weight. All the aboveground and underground parts were separately ground into powders with an agate mortar, sieved through a 100 mesh nylon sieve, and kept in Teflon bags until test. Soil samples were air dried in the laboratory, with biological debris, plant roots and leaves, and gravels excluded, ground and sieved through a 100 mesh nylon sieve, and then kept in Teflon bags.

The total amount of Sb and As in soil and vegetable parts was obtained with the method of USEPA-3050B. For the bioaccessibility tests of Sb and As, SBET (Simplified Bioaccessibility Extraction Test) is opted [19, 22]. First, 0.5 grams of edible vegetable parts (the edible parts of* Raphanus sativus* L. and* Daucus carota* L. are the underground, while the edible parts of other vegetables are aboveground) was placed in a centrifuge tube and mixed with 25 mL of amino acid solution (0.4 mol L^−1^, pH = 1.5 preadjusted with concentrated hydrochloric acid). This mixture was rotated end-over-end at 30 rpm for 1 hour at 37°C, centrifuged at high speed of 10000 r min^−1^ for 15 minutes in a high speed centrifuge (TGL16 M, Hunan Kaida, China), then filtered through a polyethylene syringe filter with a pore size of 0.2 *μ*m, and diluted to 50 mL. The content of Sb and As in all the samples was measured with Hydride Generation-Atomic Fluorescence Spectrometry (AFS-9700, Beijing Haiguang, China). All the parameters were listed in Table 1. Both Sb and As calibration curves showed good linearity (*r* > 0.999). The reagents used in sample processing and analysis were analytical reagents, from Sinopharm Chemical Reagent Co., Shanghai, China.

The relationship between the heavy metals contained in vegetables and those in soils can be calculated via bioaccumulation coefficient (BAC) and biotransfer coefficient (BTC) as follows:

(1) BAC=CuCs,BTC=CaCu,

wherein *C*_*u*_, *C*_*a*_, and *C*_*s*_ represent the heavy metal concentration in underground and aboveground parts of vegetables and soil (mg kg^−1^), respectively.

### 2.4. Quality Control and Statistical Analysis

To assure the reliability of the test results, the standard reference samples of soil (GBW07406) and vegetable (GBW10015 (GSB-6)) obtained from the National Institute of Metrology were also digested in the same way with the collected samples of soils and vegetables. The test results of the reference standard samples were listed in Table 2. Meanwhile, in the test of collected soil and vegetable samples, reagent blank determinations were used, and 10% of all the samples were retested; the relative standard difference (RSD) between the two tests should be lower than 10%.

The statistical package SPSS 17.0 was used for data analysis of Sb and As concentration in soils and vegetables. Statistical description was made on data of Sb and As concentration in 60 soil samples and 60 vegetable samples of 10 species with one-way ANOVA; correlation between Sb, As in soils and Sb, As in vegetables was determined using bivariate analysis. A significance level of *P* < 0.05 was used throughout the study.

### 2.5. Health Risks to Residents from Heavy Metals in Vegetables

The previous studies usually examined the human consumption of water, food, and air to assess the health risks to residents around XKS Sb mine brought by the consumption of vegetables [20, 23]. This research, however, based on the previous studies, employed the quotient of CDI (chronic daily intake) to assess the health risks to residents from Sb and As in vegetables. It can be calculated using the following formula:

(2) $$\begin{array}{c} \text{CDI}=\frac{C\times \text{IR}\times \text{ED}\times \text{EF}\times F_{d}}{\text{BW}\times \text{AT}\times 365}, \end{array}$$

wherein CDI stands for chronic daily intake (mg kg^−1^ d^−1^), *C* is the concentration of heavy metals (Sb and As) in vegetables that may be absorbed by humans (mg kg^−1^), IR is the intake rate, per capita consumption of vegetables (0.38 kg d^−1^) [20], ED stands for exposure duration (25 years) [24], EF is exposed frequency (350 d year^−1^) [24], *F*_*d*_ is the ratio of vegetable fresh weight converted to dry weight (0.1) [25], BW is the average body weight (61.8 kg) [26], and AT is the average exposure time, assuming 72 years in this study [27]. RfD means reference dose, an index provided by the US Environmental Protection Agency [28] and the WHO [29] for the evaluation of toxic pollutants; the RfD values of Sb and As are 4 × 10^−4 ^mg kg^−1^ d^−1^ and 3 × 10^−4^ mg kg^−1^ d^−1^. The health risks from consumption of metal contaminated vegetables were assessed based on the hazard quotient (HQ), which is calculated as follows:

(3) $$\begin{array}{c} \text{HQ}=\frac{\text{CDI}}{\text{RfD}}. \end{array}$$

If the value of HQ is lower than 1, it shows that the residents have no obvious health risks; if the value of HQ is equal to or higher than 1, it indicates that the residents have relatively high health risks.

## 3. Results and Discussion

### 3.1. Analysis of Concentrations of Sb and As in XKS Mine Soils

Concentrations of Sb and As in the 60 soil samples from the four sampling sites were between 143.74~3947.68 mg kg^−1^ and 13.69~255.51 mg kg^−1^, respectively, and their mean values were 1267.20 mg kg^−1^ and 94.44 mg kg^−1^, respectively. These values were in accordance with those reported previously [12, 14, 30].

The background concentrations of Sb and As in soils are 0.3~8.4 mg kg^−1^ [31] and 5~10 mg kg^−1^ [32] for the Earth, 1.06 mg kg^−1^ [33] and 9.2 mg kg^−1^ [34] for China, and 2.98 mg kg^−1^ and 14 mg kg^−1^ for Hunan [35], respectively. The concentrations of Sb and As in soils surrounding XKS mine were, respectively, 150 times, 1195 times, 425 times, 9.4 times, 10 times, and 6.7 times those of the Earth, China, and Hunan, much higher than the maximum value of 36 mg kg^−1^ and 8 mg kg^−1^ set by WHO. The results indicated that the soils around the XKS mine are seriously contaminated by Sb and As.

Meanwhile, the correlation analysis of the two variables showed that Sb and As in soils around the mine have significant positive correlation (*r* = 0.755, *P* < 0.05, *n* = 60), indicating that the two elements have a high homology. This may be caused by the fact that the soil contaminations of Sb and As are from mining, smelting, and tailings dust and that Sb and As are closely associated congeners, having similar chemical properties as well as similar geochemical behavior [36].

**Figure 2:**
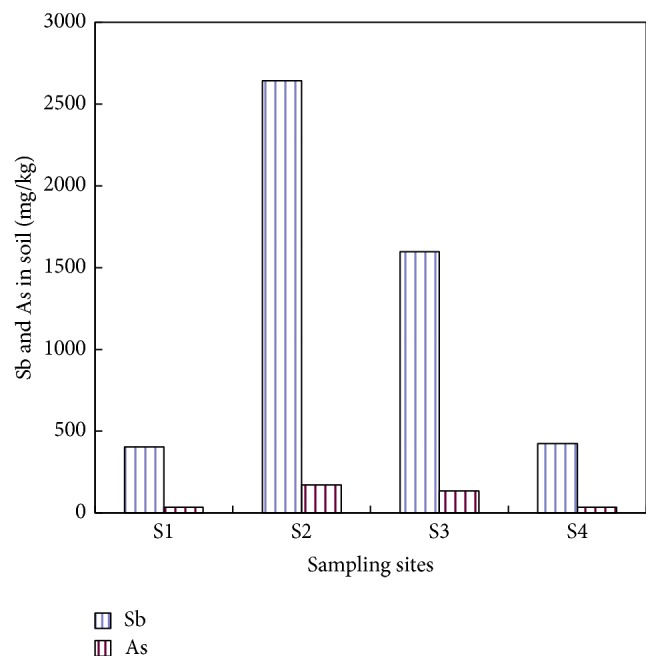
Sb and As distribution in soil of four sampling sites.

The ANOVA analysis of the concentrations of Sb and As in soils from the four sampling sites showed significant differences (*F*_Sb_ = 128.56, *P*_Sb_ = 0.00; *F*_As_ = 90.83, *P*_As_ = 0.00). The mean values of Sb and As soil concentrations in the four sampling sites (S1, S2, S3, and S4) were shown in Figure 2. The average Sb concentrations of the four sampling sites were 405.04 mg kg^−1^, 2641.55 mg kg^−1^, 1596.57 mg kg^−1^, and 425.67 mg kg^−1^, respectively, while the average As concentrations were 34.86 mg kg^−1^, 172.44 mg kg^−1^, 135.19 mg kg^−1^, and 35.27 mg kg^−1^. The extent of contaminations of Sb and As in soils formed such an order as S2 > S3 > S4 > S1. Sampling site S2, located near the South mine waste-rock piles and smelting slag piles, is most serious in Sb and As pollution, which may be coinfluenced by smelting dust, rain leaching of mining waste-rock and smelting slag, and mining waste water. Sampling site S3, located near the tailings dam, also has a higher concentration of Sb and As in soils, partly because of the dry and wet deposition of tailings dust as well as the mining waste water. Sampling sites S1 and S4 have relatively lighter contaminations of Sb and As in soils; the reasons may be that S1 is 2.0 km away from the North mine, with its location being higher than the North mine, and S4 is much far away from the mine, but local residents irrigated vegetables with the water from Lianxi River that was contaminated by mining and smelting, so its concentration of Sb and As in soil is similar to that of S1.

This suggests that mining and smelting activities in XKS mine have caused serious pollution to the surrounding soils. The less the distance away from the mine, the more severe the contamination.

### 3.2. Distribution of Sb and As in Vegetables from Various Sampling Sites

**Figure 3:**
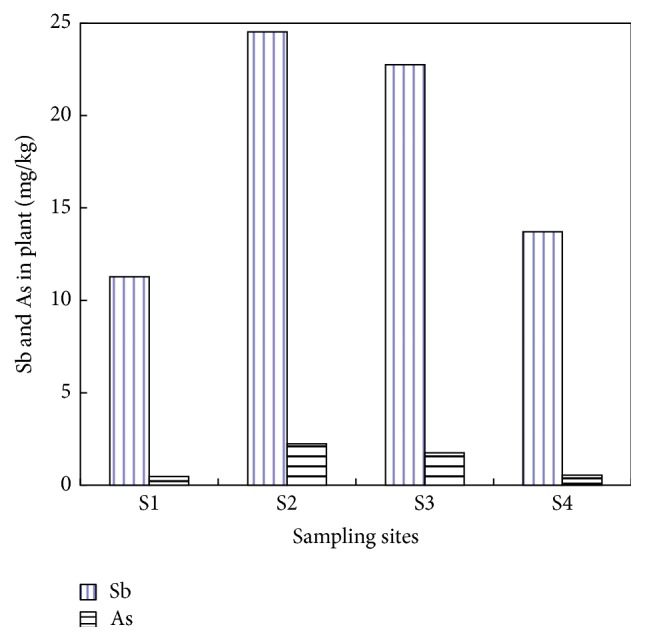
Mean concentration of Sb and As in vegetables collected in different sites.

Residents surrounding XKS mine consume the vegetables they grow there. A total of 60 vegetable samples of 10 species were collected from the four sampling sites. The vegetable samples from the same sampling site formed an entity. The concentrations of Sb and As in vegetables from the four sites were shown in Figure 3. The ANOVA analysis indicated that the underground parts of vegetables from the four sampling sites showed a significant difference (*F*_Sb_ = 6.52, *P*_Sb_ = 0.001; *F*_As_ = 30.84, *P*_As_ = 0.000). Vegetables in S2 had the highest average concentrations of Sb (24.53 mg kg^−1^) and As (2.23 mg kg^−1^), and vegetables in S1 had the lowest average concentration of Sb (11.27 mg kg^−1^) and As (0.47 mg kg^−1^), which corresponded, respectively, to the Sb and As concentrations in soils of S2 and S1. Correlation studies showed that Sb and As in the underground parts of vegetables and in soils from the four sampling sites had significant correlation (*r*_Sb_ = 0.52, *P*_Sb_ < 0.05; *r*_As_ = 0.81, *P*_As_ < 0.05).

### 3.3. Concentration Distribution and Accumulation of Sb and As in Different Vegetables

The plants grown in the uncontaminated soils have a background concentration of Sb and As ranging between 2–50 *μ*g kg^−1^ [37, 38] and 0.01~1.5 mg kg^−1^ [39], respectively. The concentrations of Sb and As in different plants vary, depending on the nature of the soils and the migration of Sb and As in contaminated soils [6, 40]. Studies have shown that plants with Sb concentration of 5–10 mg kg^−1^ are of toxicity [41], but He [30] pointed out that plants with the Sb concentration of 5 mg kg^−1^ would be toxic and that it would stimulate the growth of plants if the As concentration is less than 10 mg kg^−1^, while it would toxicate the plants if over 50 mg kg^−1^ [42]. The 60 vegetable samples of 10 species collected from XKS mine had average concentrations of Sb and As of 6.65 mg kg^−1^~29.31 mg kg^−1^ and 0.61 mg kg^−1^~1.73 mg kg^−1^ respectively, indicating that Sb in soils exerts a certain inhibition on vegetable growth, but As has little effect on vegetable growth.

The heavy metal concentrations in different parts of vegetables showed great differences [30]. The concentrations of Sb and As in the collected 10 species of vegetables were in Table 3. As shown in Table 3, the underground part of* Daucus carota* L. had the highest Sb concentration (mean value: 23.72 mg kg^−1^), and the underground part of* Allium fistulosum* L. had the highest As concentration (mean value: 2.08 mg kg^−1^). The underground part of* Brassica pekinensis* L. had the lowest Sb and As concentrations (mean value: 7.76 mg kg^−1^ and 0.60 mg kg^−1^, resp.) and the aboveground part of* Raphanus sativus* L. had the highest Sb concentration (mean value: 37.39 mg kg^−1^). The aboveground part of* Coriandrum sativum* L. had the highest As concentration (mean value: 2.06 mg kg^−1^) and the aboveground part of* Brassica pekinensis* L. had the lowest Sb and As concentrations (mean value: 5.85 mg kg^−1^ and 0.63 mg kg^−1^, resp.).

**Figure 4:**
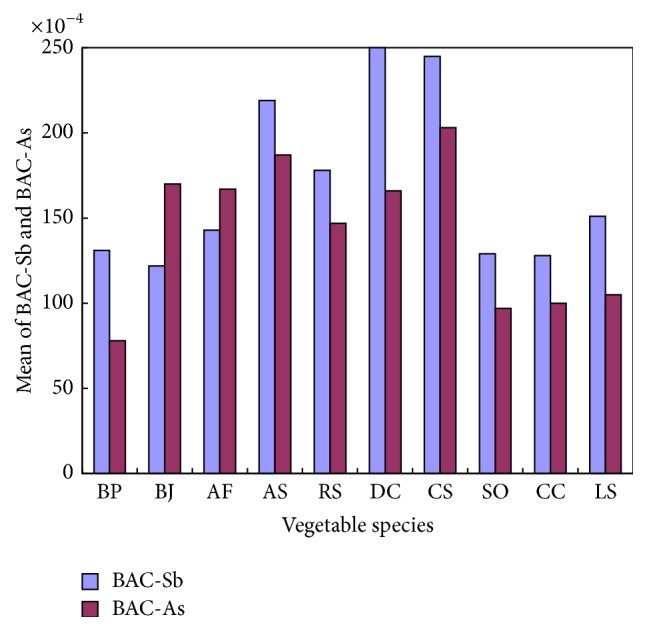
Mean BAC of Sb and As in 10 species.

Vegetables had different cumulative capacity of Sb and As. The differences of Sb and As concentration in soils can be eliminated via BAC calculation, and the mean bioaccumulation coefficients (BAC) of the 10 species of vegetables were obtained in Figure 4. As can be seen from the figure, the average BAC_Sb_ and BAC_As_ values of all vegetables were less than 0.03, indicating that the bioavailability of Sb and As in soils from the four sampling sites is very low. As for the vegetables,* Daucus carota* L. had a higher value of BAC_Sb_ (0.0250), indicating that* Daucus carota* L. has advantages over others in absorbing Sb from soils, and* Coriandrum sativum* L. had a higher value of BAC_As_ (0.0203), indicating that it has a strong ability in absorbing As from soils.

**Figure 5:**
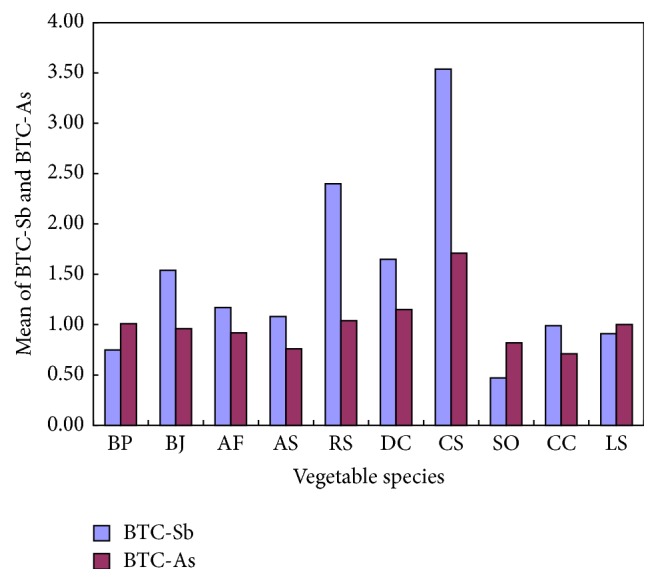
Mean BTC of Sb and As in 10 species.

Concentrations of Sb and As were not uniform in different parts of certain plants. For example, the 19 kinds of vegetables and crops such as carrots, onions, and tomatoes had the Sb concentrations of 0.02–0.09 mg kg^−1^ in the underground part, but they had Sb concentrations of 0.02–2.2 mg kg^−1^ in stems and leaves [23].* Mentha aquatica* had an As concentration of 540 mg kg^−1^ in root but had As concentration of 216 mg kg^−1^ in leaves [43]. BTC can be used to evaluate the distribution of Sb and As in the underground and aboveground parts of the plants. The higher the values of the BTC are, the more efficiently the heavy metals are transferred to the aboveground parts through the roots [44]. The mean values of BTC_Sb_ and BTC_As_ of vegetable samples were shown in Figure 5. As indicated in Figure 5, among the 10 species of vegetables, the mean values of BTC_Sb_ and BTC_As_ of* Raphanus sativus* L.,* Daucus carota* L., and* Coriandrum sativum* L. were higher than 1.0. In particular, the mean values of BTC_Sb_ and BTC_As_ of* Coriandrum sativum* L. were even up to 3.54 and 1.71, respectively. The results indicated that the mentioned three vegetables have a strong ability in transferring Sb and As aboveground through the roots; that is to say, the three species of vegetables have a strong redistributive capacity of Sb and As.

### 3.4. Assessment of Health Risks Caused by Sb and As

The previous researches calculated the HQ and CDI values of heavy metals from food intake on the assumption that the heavy metals contained in vegetables were 100% absorbed by human beings [20, 45], which obviously unscientifically enlarged the health risk.

In fact, instead of being completely absorbed by the body, a large proportion of heavy metals were excreted out [46, 47], so the method of SBET is practical to calculate the availability of Sb and As in the edible vegetable parts. The SBET values of Sb and As in the edible parts of 10 species of vegetables were in Table 3. The calculation results of chronic daily intake (CDI) and hazard quotient (HQ) of Sb and As that residents in the four sampling sites around XKS mine absorbed were shown in Table 4.

As can be seen from Table 4, CDI_Sb_ of the residents in the sampling areas is higher than the limit (RfD_Sb_ = 4 × 10^−4^ mg kg^−1^ d^−1^) recommended by USEPA and WHO, with CDI_Sb_ in S2 being the highest (13.31 × 10^−4 ^mg kg^−1^ d^−1^); CDI_As_ is lower than the recommended limit (RfD_As_ = 3 × 10^−4^ mg kg^−1^ d^−1^). The average chronic daily intake of Sb is significantly greater than that of As.

Hazard quotient (HQ) is a useful parameter to assess health risks brought by the consumption of food contaminated with heavy metals [48]. The HQ_Sb_ index values of the four sampling areas were all higher than 1.0 (1.61, 3.33, 2.45, and 1.78, resp.), indicating that the vegetables have obvious health risks on human health; HQ_As_ was lower than 0.4, indicating that As in vegetables brings less health risks to people than Sb.

According to the research, the heavy metal Sb in vegetables brings higher HQ to the local residents than As. Therefore, it is highly important to take appropriate measures to control the heavy metal contamination in soils around XKS mine and to bring the accumulation of heavy metals in the edible parts of vegetables to a safe level.

## 4. Conclusions

From the results of the soil and vegetable samples collected, we came to a better knowledge that mining and smelting of Sb greatly impacted the surrounding environment and brought potential risks to people's health. The contamination of Sb and As in soils of the four sampling sites formed such an order, S2 > S3 > S1 > S4, which indicates that the contamination of Sb and As in soils diminishes with distance from the mine and slag piles. The concentration of Sb and As in vegetable samples from the four sampling sites varied. Among the 10 species of vegetables,* Coriandrum sativum* L. has the highest concentration of Sb, and* Allium fistulosum* L. has the highest concentration of As, while* Brassica pekinensis* L. has the lowest concentration of both Sb and As. The accumulations of Sb and As in various vegetables were narrowly different (both BAC_Sb_ and BAC_As_ were less than 0.03), but the distribution of Sb and As in various vegetables was severely different (especially* Coriandrum sativum* L., BTC_Sb_ = 3.54, BTC_As_ = 1.71). The calculation of CDI and HQ showed that the health risks to local residents caused by Sb in vegetables are much higher than As. Therefore, effective measures must be taken to control the toxic heavy metal pollution, especially the pollution caused by Sb in the surrounding areas of XKS mine.

## Figures and Tables

**Table 1:** Instrumental and operative conditions of AFS-9700.

Instrument parameters	Sb	As
Photomultiplier voltage	290 V	300 V
Height of atomiser	8 mm	8 mm
Flow of carrier gas (Ar)	300 mL min^−1^	300 mL min^−1^
Lamp current	70 mA	50 mA
Flow of sheath gas (Ar)	900 mL min^−1^	900 mL min^−1^
Reading time	12 s	15 s
Delay time	1 s	1 s
Current carrying	5% HCl	5% HCl
Reaction liquid	2% KBH_4_ + 0.5% NaOH	2% KBH_4_ + 0.5% NaOH
Measure method	Standard curve method	Standard curve method
Reading method	Peak area	Peak area

**Table 2:** Summary of measures of certified reference element concentration (mg kg^−1^, mean ± SD, *n* = 3) in CRMs.

	GBW07406			GBW10015 (GSB-6)		
	Certified value	Measured value	Recovery^a^ (%)	Certified value	Measured value	Recovery (%)
Sb	60 ± 10	58 ± 1.5	97	0.043 ± 0.014	0.040 ± 0.004	93
As	220 ± 21	210 ± 6.5	95	0.23 ± 0.03	0.22 ± 0.01	96

Note: ^a^values quoted on dry weight basis; recovery (%) = (mean measured value/mean certified value) × 100%.

**Table 3:** Sb and As concentrations in different vegetables and SBET values of edible vegetable parts (mg kg^−1^).

Vegetable	Total		Underground		Aboveground		SBET	
	Sb	As	Sb	As	Sb	As	SBET_Sb_	SBET_As_
BP	3.34–13.56 (6.65)	0.16–1.61 (0.61)	4.10–15.74 (7.76)	0.21–1.48 (0.60)	2.64–12.29 (5.85)	0.17–1.77 (0.63)	0.64–2.58 (1.21)	0.018–0.261 (0.087)
BL	9.97–25.94 (17.10)	0.58–2.49 (1.63)	7.32–22.86 (13.07)	0.78–2.23 (1.64)	11.44–28.46 (19.28)	0.43–2.72 (1.65)	3.21–8.25 (5.19)	0.086–0.597 (0.370)
AF	10.32–22.87 (16.62)	0.46–4.11 (1.95)	8.84–22.07 (15.00)	0.53–4.85 (2.08)	10.66–23.29 (17.08)	0.42–3.82 (1.90)	3.39–5.67 (4.60)	0.128–1.468 (0.709)
AS	6.53–44.48 (24.86)	0.32–2.02 (1.11)	7.03–37.89 (22.60)	0.35–2.67 (1.46)	6.30–46.34 (25.37)	0.31–1.81 (1.00)	2.47–15.79 (8.53)	0.111–0.679 (0.335)
RS	7.42–31.39 (22.39)	0.42–2.41 (1.35)	5.59–21.52 (15.05)	0.39–2.55 (1.39)	13.04–57.92 (37.39)	0.47–2.07 (1.32)	0.78–2.58 (1.91)	0.033–0.245 (0.123)
DC	7.75–45.27 (28.01)	0.23–2.88 (1.49)	5.14–41.05 (23.72)	0.21–2.84 (1.45)	10.91–58.35 (37.06)	0.32–2.97 (1.54)	0.58–3.73 (2.31)	0.018–0.202 (0.114)
CS	18.65–39.28 (29.31)	0.43–3.84 (1.73)	5.51–15.87 (10.86)	0.29–2.59 (1.23)	24.61–50.23 (36.78)	0.52–4.36 (2.06)	7.19–15.94 (11.31)	0.230–1.657 (0.783)
SO	4.47–21.86 (11.38)	0.28–1.82 (0.98)	6.25–29.30 (18.46)	0.34–1.92 (1.12)	2.97–18.20 (8.65)	0.25–1.76 (0.92)	1.21–7.12 (3.32)	0.063–0.554 (0.257)
CC	5.20–23.92 (12.04)	0.20–1.81 (0.85)	4.85–17.89 (11.76)	0.23–2.30 (1.08)	5.31–26.28 (12.11)	0.19–1.58 (0.71)	1.27–6.01 (2.67)	0.066–0.412 (0.209)
LS	7.20–19.65 (12.25)	0.33–1.41 (0.82)	7.05–21.48 (13.48)	0.37–1.27 (0.80)	7.28–18.74 (11.53)	0.31–1.46 (0.83)	2.27–5.84 (3.72)	0.059–0.361 (0.206)

( ): average value.

**Table 4:** Daily dietary intake of Sb and As from vegetables and health risk index.

Sampling sites	Heavy metals	CDI (mg kg^−1^ d^−1^)	HQ
S1	Sb	6.43 × 10^−4^	1.61
	As	0.26 × 10^−4^	0.09

S2	Sb	13.31 × 10^−4^	3.33
	As	1.18 × 10^−4^	0.39

S3	Sb	9.81 × 10^−4^	2.45
	As	0.88 × 10^−4^	0.29

S4	Sb	7.10 × 10^−4^	1.78
	As	0.31 × 10^−4^	0.10
